# Conservation between higher plants and the moss *Physcomitrella patens *in response to the phytohormone abscisic acid: a proteomics analysis

**DOI:** 10.1186/1471-2229-10-192

**Published:** 2010-08-27

**Authors:** Xiaoqin Wang, Tingyun Kuang, Yikun He

**Affiliations:** 1 College of Life Sciences, Capital Normal University, Beijing 100048, China; 2 Key Laboratory of Urban Agriculture (North) of Ministry of Agriculture P. R. China, Beijing 102206, China; 3 Beijing University of Agriculture, Beijing 102206, China; 4 Department of Biology, Washington University in St. Louis, MO 63130, US

## Abstract

**Background:**

The plant hormone abscisic acid (ABA) is ubiquitous among land plants where it plays an important role in plant growth and development. In seeds, ABA induces embryogenesis and seed maturation as well as seed dormancy and germination. In vegetative tissues, ABA is a necessary mediator in the triggering of many of the physiological and molecular adaptive responses of the plant to adverse environmental conditions, such as desiccation, salt and cold.

**Results:**

In this study, we investigated the influence of abscisic acid (ABA) on *Physcomitrella patens *at the level of the proteome using two-dimensional gel electrophoresis (2-DE) and liquid chromatography-tandem mass spectrometry (LC-MS/MS). Sixty-five protein spots showed changes in response to ABA treatment. Among them, thirteen protein spots were down-regulated; fifty-two protein spots were up-regulated including four protein spots which were newly induced. These proteins were involved in various functions, including material and energy metabolism, defense, protein destination and storage, transcription, signal transduction, cell growth/division, transport, and cytoskeleton. Specifically, most of the up-regulated proteins functioned as molecular chaperones, transcriptional regulators, and defense proteins. Detailed analysis of these up-regulated proteins showed that ABA could trigger stress and defense responses and protect plants from oxidative damage. Otherwise, three protein kinases involved in signal pathways were up-regulated suggesting that *P. patens *is sensitive to exogenous ABA. The down-regulated of the Rubisco small subunit, photosystem II oxygen-evolving complex proteins and photosystem assembly protein ycf3 indicated that photosynthesis of *P. patens *was inhibited by ABA treatment.

**Conclusion:**

Proteome analysis techniques have been applied as a direct, effective, and reliable tool in differential protein expressions. Sixty-five protein spots showed differences in accumulation levels as a result of treatment with ABA. Detailed analysis these protein functions showed that physiological and molecular responses to the plant hormone ABA appear to be conserved among higher plant species and bryophytes.

## Background

Plants undergo continuous exposure to various biotic and abiotic stresses in their natural environment. To survive under such conditions, plants activate signaling cascades that lead to the accumulation of endogenous hormones which, in turn, trigger the induction of defense responses. Abscisic acid (ABA) is a small, lipophilic phytohormone that regulates many important aspects of plant growth and development and plays a crucial role in cellular responses to environmental stresses such as drought, cold, salt, wounding, UV radiation, and pathogen attack [1]. It is ubiquitous in lower and higher plants and has also been found in algae [2], fungi [3], and even mammalian brain tissue [4].

Many studies have been carried out that characterize ABA signaling pathways and physiological responses to ABA in higher plants. Mutational analyses in Arabidopsis have led to the identification of several genes that are involved in ABA signaling pathways. The proteins involved in ABA signaling pathways include G proteins, protein phosphatases, and protein kinases [5]. Target proteins that respond to ABA can be directly involved in cellular protection or can act as transcriptional factors that link extracellular signaling to the regulation of transcription in eukaryotic cells an important part of the physiological response to ABA is achieved through gene expression and protein synthesis. Transcriptome analyses have shown that ABA dramatically alters gene expression. More than 1300 ABA-regulated genes were identified by random mass sequencing of *Arabidopsis *transcripts [6,7]. Proteome research has also indicated that exogenous ABA can induce the synthesis of many proteins in rice seedlings. These proteins are involved in signaling pathways, transcription, cell growth and division, photosynthesis, protein synthesis and trafficking, and defense/stress-response, among others [8]. To date however, relatively little is known regarding ABA signaling pathways in bryophytes.

Bryophytes were the first land plants and they have been a powerful experimental tool and model for the elucidation of complex biological processes [9-11]. Evolutionary studies have indicated that bryophytes may form a sister clade with tracheophytes (vascular plants). Analysis of organisms from these ancient clades can make significant contributions to understanding the development [12], physiology [13], phylogenetics [14] and stress-induced cellular responses among plants [15]. Recently, comparison of the draft genome sequence of *P. patens *with the genomes of flowering plants revealed evolutionary insights into the conquest of land by plants. Furthermore, bryophyte physiology is regulated by many of the same phytohormones used by higher plants (e.g., auxins, abscisic acid, and gibberellins) and uses similar mechanisms of intracellular messaging (e.g. Ca2+) found in higher plants [13,16,17].

In recent years, the moss *P. patens *become a new model for studying the phytohormone ABA. ABA is involved in abiotic stress adaptation of *P. patens*, since exogenously applied ABA increases freezing tolerance [18] and increased endogenous ABA levels are detected upon osmotic stress treatment [19]. Moreover, plants like *P. patens *comprising both protonemata and gametophores will tolerate complete desiccation following slow drying, if first pretreated with ABA, a process that is associated with a substantial increase in intracellular levels of sucrose [20] as is cold acclimation leading to freezing tolerance [21]. ABA-induced stress tolerance in *P. patens *is also accompanied by increased expression of stress-related genes [6,7]. However, the role of ABA or the signaling pathways in response to environmental stresses in the moss *P. patens *has yet to be elucidated.

Here, proteomics has been used to increase understanding of ABA signaling pathways and defense responses in *P. patens*. We have examined the global changes of proteins in *P. patens *following ABA treatment using two dimensional gel electrophoresis (2-DE) coupled with LC-MS/MS. Sixty-five proteins have been identified as being up- or down-regulated in response to ABA treatment. These proteins are involved in metabolism and energy, defense responses, protein trafficking and storage, transcription, and signaling pathways, as well as others. Our study reveals the complex signaling pathways and defense responses by which ABA functions in *P. patens*. It also suggests that molecular responses to ABA may be highly conserved between higher plant species and bryophytes.

## Results

### Proteome profile changes in *P. patens *under ABA treatment

**Figure 1:**
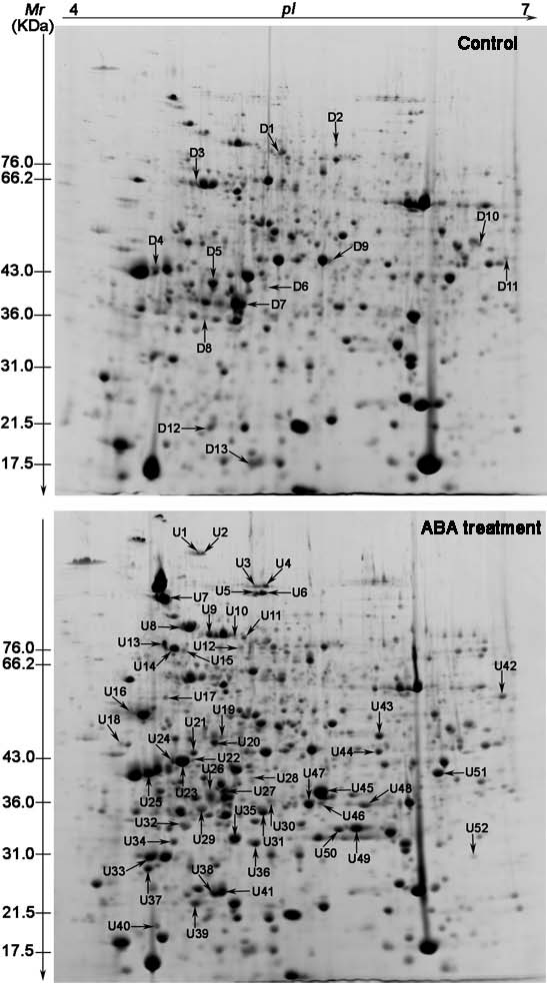
**Comparison of the proteome pattern of *P. patens *gamatophores under control and ABA treatments**. The arrows indicate the ABA-responsive proteins. Down-regulated proteins (spots D1-D13) are indicated in the control image. Up-regulated proteins and newly-induced proteins (spots U1-U52) are indicated in the image following 50 μM ABA treatment.

**Figure 2:**
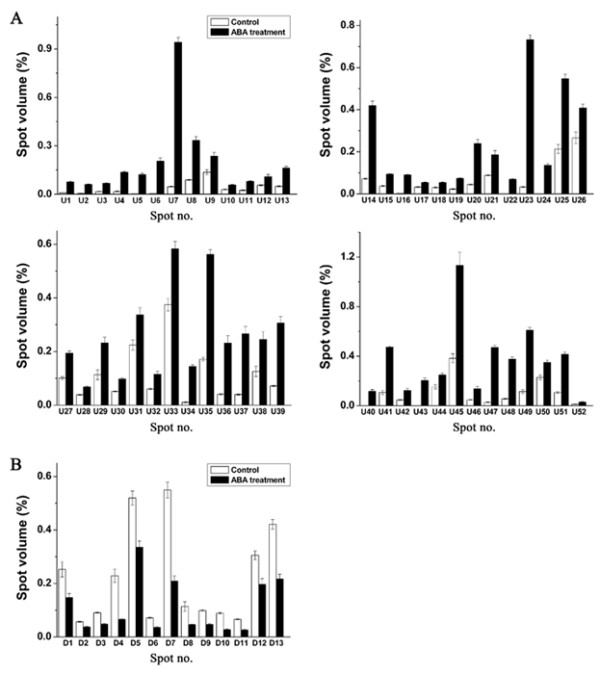
**Relative abundances (%) of individual ABA-responsive proteins before and after ABA treatment**. **A**, Proteins up-regulated in response to ABA as identified in Figure 1; **B**, Proteins down-regulated in response to ABA as identified in Figure 1. The protein abundance is presented as the percentage of the total spot volume associated with each identified spot.

Changes in the proteome profile between control and ABA treated plants suggested that exogenous ABA could trigger one or more responses in *P. patens*. In order to ensure statistically results, the experiments were carried out in triplicate. After CBB R-250 staining, more than 1,300 protein spots could be detected for each sample. Representative gels from control and ABA-treated plants and proteins showing altered abundance are presented in Figure 1. Two-dimensional images were analyzed using ImageMaster™ 2D Platinum software. The volume percentage of each spot was estimated and compared across the gels. In the samples following ABA treatment, we repeatedly analyzed 89 protein spots, whose relative abundance was at least 1.5-fold different from the control. The proteins associated with 65 of these protein spots were subsequently identified using LC-MS/MS. Among them, 13 protein spots (D1-D13) were down-regulated, 52 protein spots (U1-U52) were up-regulated by ABA treatment including four protein spots (U22, U24, U40, U43) which were only present in the ABA-treated samples (Figure 1). Quantitative analysis indicated that the abundance of these proteins changed [see Additional File 1: Supplemental Table S1] in response to ABA treatment (Figure 2) suggesting that different physiological and biochemical processes are modified following ABA treatment.

### Protein identification by LC-MS/MS

The protein spots of interest were excised from preparative 2-D gels. After trypsin autolysis, the digested spots were analyzed by LC-MS/MS. The resulting amino acid sequences were searched against databases using SEQEST software. As the *P. patens *sequence information is limited, there were some samples that did not yield significant matches. For analysis of these samples, the Arabidopsis database was used. Previous transcriptome research has shown that the gene set in *P. patens *is very similar to that of *A. thaliana *[22]. The results of the database search are listed in Table 1 and Supplemental Table S2 [see Additional File 2: Supplemental Table S2].

**Table 1:** Identification of ABA-responsive proteins in *P. patens*

Spot **no**.	**Accession no**.	**Theor./exp**. Mass (kDa)	Theor./exp. pI	Description	NMP	SC (%)	Species
D1	CAA68141.1	76.1/77.8	5.83/5.44	Chloroplast FtsH protease	6	16.22	*A. thaliana*
D2	CAB79006.1	127.3/81.2	7.92/5.81	Hypothetical protein	1	1.59	*A. thaliana*
D3	BAC85045.1	53.3/68.3	4.98/4.88	ATP synthase beta subunit	18	70.04	*P. patens*
D4	CAB42911.1	35.0/45.7	5.92/4.61	Putative protein 1 photosystem II oxygen-evolving complex	2	8.76	*A. thaliana*
D5	E86427	57.4/42.6	7.63/4.99	Hypothetical protein T4k22.7	1	2.95	*A. thaliana*
D6	P46875	85.0/41.6	5.9/5.35	Kinesin-3(Kinesin-like protein C)	1	1.72	*A. thaliana*
D7	CAC43717.1	49.7/38.4	6.09/5.19	Unnamed protein product	2	6.74	*P. patens*
D8	CAC43713.1	31.9/36.1	5.36/4.94	Unnamed protein product	10	40.98	*P. patens*
D9	BAC87878.1	21.1/47.2	8.77/5.77	Rubisco small subunit	5	45.65	*P. patens*
D10	CAB80784.1	55.7/50.9	9.0/6.77	AT4g00260	1	4.49	*A. thaliana*
D11	CAB80829.1	40.9/47.9	8.41/6.94	AT4g04640	1	3.49	*A. thaliana*
D12	NP_904201.1	19.6/21.7	4.97/4.97	Photosystem assembly protein ycf3	6	65.48	*P. patens*
D13	BAA83481.1	23.3/18.4	8.74/5.24	Rubisco small subunit	10	49.53	*P. patens*
U1	CAA11281.1	93.9/116.8	6.48/4.91	Stelar K^+ ^outward rectifying channel	1	1.45	*A. thaliana*
U2	F96615	90.8/116.8	9.75/4.94	Probable Myb-family transcription fator	1	2.06	*A. thaliana*
U3	AAQ88112.1	41.7/100.5	5.31/5.28	Actin	9	37.83	*P. patens*
U4	CAB80488.1	54.3/100.5	5.59/5.34	Calcium-dependent protein kinase-like protein	1	2.07	*A. thaliana*
U5	CAC03450.1	44.1/96.3	5.78/5.27	Ser/thr specific protein kinase-like protein	1	3.09	*A. thaliana*
U6	D84501	13.21/97.2	6.75/5.33	Hypothetical protein At2g12170	1	10.43	*A. thaliana*
U7	CAB79338.1	76.5/94.7	5.07/4.69	HSP 70-like protein	3	5.57	*A. thaliana*
U8	CAB79338.1	76.5/83.0	5.07/4.80	HSP 70-like protein	3	5.57	*A. thaliana*
U9	P22954	71.4/79.7	5.03/4.98	Heat shock cognate 70 kDa protein 2	12	24.81	*A. thaliana*
U10	CAA05547.1	71.1/79.7	5.14/5.15	Heat shock protein 70	10	12.92	*A. thaliana*
U11	CAA05547.1	71.1/78.8	5.14/5.21	Heat shock protein 70	11	19.69	*A. thaliana*
U12	CAB87667.1	82.1/74.8	6.21/5.17	Subtilisin-like protease-like protein	1	1.85	*A. thaliana*
U13	AAM28648.1	56.6/76.8	4.85/4.68	Protein disulfide isomerase-like protein	11	27.15	*P. patens*
U14	CAB80574.1	96.5/75.1	5.76/4.76	Putative receptor-like protein kinase	1	1.59	*A. thaliana*
U15	CAB89378.1	14.67/55.9	7.78/4.47	Putative proline-rich protein	1	16.92	*A. thaliana*
U16	CAB43694.1	33.7/55.0	10.08/4.55	Fibrillarin-like protein	1	6.25	*A. thaliana*
U17	BAD94943.1	40.1/48.1	5.13/4.41	AMP deaminase like protein	1	4.38	*A. thaliana*
U18	CAB77733.1	27.7/49.7	9.58/5.06	Putative expansin	1	3.53	*A. thaliana*
U19	CAB80540.1	49.1/48.3	9.71/5.02	Extensin-like protein	1	3.35	*A. thaliana*
U20	CAB54558.1	47.5/46.1	6.04/4.88	Plastid division protein ftsZ1	4	18.78	*P. patens*
U21	AAY78603.1	37.6/44.5	5.51/4.87	PfkB-type carbohydrate kinase family protein	1	3.77	*A. thaliana*
U22	Q9FH83	156.1/44.2	5.61/4.80	Probable WRKY transcription factor 52	1	4.46	*A. thaliana*
U23	CAB75445.1	35.0/44.2	5.21/4.75	Fructokinase-like protein	1	3.99	*A. thaliana*
U24	AAG61085.1	31.1/41.8	5.08/4.58	Intracellular pathogenesis-related protein-like protein	1	5.17	*P. patens*
U25	BAD94888.1	44.5/38.5	5.09/4.98	DnaK-type molecular chaperone hsc 70.1-like	2	9.41	*A. thaliana*
U26	CAB88528.1	33.4/38.5	5.245.09	bZIP transcription factor-like protein	1	4.39	*A. thaliana*
U27	CAC85344.1	38.9/40.6	8.68/5.26	Cullin 3a	1	5.33	*A. thaliana*
U28	Q39044	53.8/35.4	5.84/4.93	Vacuolar processing enzyme, beta-isozyme precursor	1	3.09	*A. thaliana*
U29	Q9LQ54	80.5/36.7	8.87/5.38	Probable disease resistance protein	1	2.73	*A. thaliana*
U30	G96756	27.9/35.0	5.23/5.32	Lipoxygenase	2	3.74	*A. thaliana*
U31	CAC43712.1	34.0/33.0	5.9/4.80	Unnamed protein product	5	17.43	*P. patens*
U32	CAC43713.1	31.9/28.5	5.36/4.51	Unnamed protein product	4	17.38	*P. patens*
U33	CAA67427.1	24.5/28.5	5.51/4.60	Thylakoid-bound ascorbate peroxidase	1	3.60	*A. thaliana*
U34	F96795	135.1/30.8	5.5/4.75	Hypothetical protein F28O16.9	1	1.31	*A. thaliana*
U35	Q9LKR3	73.6/30.4	5.08/5.14	Luminal binding protein precursor (BiP1) (AtBP1)	2	2..54	*A. thaliana*
U36	CAB43552.1	36.0/30.2	7.08/5.28	Phosphoribosyl diphosphate synthase	1	6.13	*A. thaliana*
U37	CAA66484.2	29.1/26.8	7.74/4.59	2-Cys peroxiredoxin	1	6.02	*A. thaliana*
U38	AAV65396.1	19.9/23.9	5.3/5.01	Physcomitrin	4	23.60	*P. patens*
U39	CAB78663.1	26.4/22.8	7.77/4.89	Enoyl-COA hydratase like protein	1	5.74	*A. thaliana*
U40	H96806	9.9/20.6	9.22/4.63	Unknown protein T32E8.4	1	19.35	*A. thaliana*
U41	AAV65396.1	19.9/24.1	5.3/5.06	Physcomitrin	6	38.76	*P. patens*
U42	CAB62598.1	50.4/59.9	6.36/6.88	N-hydroxycinnamoyl/benzoyltransferase-like protein	1	3.08	*A. thaliana*
U43	CAA66816.1	42.5/49.7	7.62/6.08	Glyceraldehyde-3-phosphate dehydrogenase	2	7.32	*A. thaliana*
U44	CAB87759.1	43.9/46.3	8.54/6.08	mRNA binding protein precursor-like	2	5.17	*A. thaliana*
U45	CAD38154.1	27.7/38.4	5.66/5.71	Putative ascorbate peroxidase	2	13.20	*P. patens*
U46	CAD38154.1	27.7/36.4	5.66/5.70	Putative ascorbate peroxidase	3	18.80	*P. patens*
U47	C96608	60.8/36.3	8.22/5.62	Hypothetical protein F25P12.91	1	3.83	*A. thaliana*
U48	CAI84534.1	116.4/36.1	6.84/5.99	Unnamed protein product	1	1.35	*A. thaliana*
U49	CAA73616.1	7.8/32.4	9.15/5.93	Multicatalytic endopeptidase	2	14.29	*A. thaliana*
U50	O04005	24.1/32.3	6.13/5.82	Peroxiredoxin (Thioredoxin peroxidase) (Rehydrin homolog)	1	6.02	*A. thaliana*
U51	CAB43552.1	36.0/41.9	7.08/6.46	Phosphoribosyl diphosphate synthase	1	6.13	*A. thaliana*
U52	O65719	71.1/28.4	4.97/6.69	Heat shock cognate 70 kDa protein 3	2	4.78	*A. thaliana*

D1-D13 are down-regulated proteins; U1-U52 are up-regulated, including induced proteins U22, U24, U40,U43; NMP, number of matched peptides; SC, sequence coverage

### Functional categorization of ABA-responsive proteins

**Figure 3:**
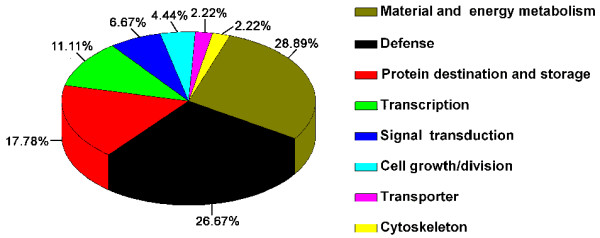
**Functional categorization of the up- and down-regulated *P. patens *proteins in response to ABA treatment**. Numerical values represent the percentage of proteins in each functional category.

The ABA-responsive proteins could be sorted into eight categories (Figure 3, Table 2) according to their biological function as described by the EU *A. thaliana *genome project [23]. These categories include material and energy metabolism (28.9%), defense (26.7%), protein synthesis and degradation (17.8%), transcription (11.1%), signal transduction (6.7%), cell growth/division (4.4%), transport (2.2%), and cytoskeleton (2.2%).

**Table 2:** Classes of proteins identified on two-dimensional gels

Protein Category	**Spot no**.
**Material and energy metabolism (28.89%)**	
ATP synthase beta subunit	D3 ↓^a^
Putative protein 1 photosystem II oxygen-evolving complex	D4 ↓
Rubisco small subunit	D9 ↓, D13 ↓
Photosystem assembly protein ycf3	D12 ↓
AMP deaminase like protein	U17 ↑^b^
PfkB-type carbohydrate kinase family protein	U21 ↑
Fructokinase-like protein	U23 ↑
Enoyl-COA hydratase like protein	U39 ↑
Glyceraldehyde-3-phosphate dehydrogenase	U43 *^c^
Multicatalytic endopeptidase	U49 ↑
Phosphoribosyl diphosphate synthase	U36 ↑,U51 ↑
Subtilisin-like protease-like protein	U12 ↑
Protein disulfide isomerase-like protein	U13 ↑
**Defense (26.67%)**	
Putative proline-rich protein	U15 ↑
Putative expansin	U18 ↑
Extensin-like protein	U19 ↑
Intracellular pathogenesis-related protein-like protein	U24 *
Disease resistance protein RPM1	U29 ↑
Lipoxygenase	U30 ↑
Thylakoid-bound ascorbate peroxidase	U33 ↑
2-Cys peroxiredoxin	U37 ↑
Physcomitrin	U38 ↑,U41 ↑
N-hydroxycinnamoyl/benzoyltransferase-like protein	U42 ↑
Putative ascorbate peroxidase	U45 ↑,U46 ↑
Peroxiredoxin	U50↑
**Protein destination and storage (17.78%)**	
Chloroplast FtsH protease	D1 ↓
HSP 70-like protein	U7↑, U8 ↑
Heat shock cognate 70 kDa protein 2	U9 ↑
Heat shock protein 70	U10 ↑, U11 ↑
DnaK-type molecular chaperone hsc 70.1-like	U25 ↑
Cullin 3a	U27 ↑
Luminal binding protein precursor (BiP1) (AtBP1)	U35 ↑
Heat shock cognate 70 kDa protein 3	U52 ↑
**Transcription (11.11%)**	
Probable Myb-family transcription fator	U2 ↑
Fibrillarin-like protein	U16 ↑
Probable WRKY transcription factor 52	U22 *
bZIP transcription factor-like protein	U26 ↑
mRNA binding protein precursor-like	U44↑
**Signal transduction (6.67%)**	
Calcium-dependent protein kinase-like protein	U4 ↑
Ser/thr specific protein kinase-like protein	U5 ↑
Putative receptor-like protein kinase	U14 ↑
**Cell growth/division (4.44%)**	
Plastid division protein ftsZ1	U20↑
Vacuolar processing enzyme, beta-isozyme precursor	U28↑
**Transporter (2.22%)**	
Stelar K^+ ^outward rectifying channel	U1↑
**Cytoskeleton (2.22%)**	
Actin	U3↑

^a ^The downward arrow represents the down-regulated protein spot on the 2-D gel;

^b ^The upward arrow represents the up-regulated protein spot on the 2-D gel;

^C ^The * represents the newly induced protein spot on the 2-D gel.

Within the material and energy metabolism category, ATP synthase beta subunit (spot D3), putative protein 1 photosystem II oxygen-evolving complex (spot D4), rubisco small subunit (spots D9, D13) and photosystem assembly protein ycf3 (spot D12) were down-regulated, and another nine proteins were up-regulated. All proteins within the defense category were up-regulated. These proteins include putative proline-rich protein (spot U15), putative expansin (spot U18), extensin-like protein (spot U19), intracellular pathogenesis-related protein-like protein (spot U24), disease resistance protein RPM1 (spot U29), lipoxygenase (spot U30), thylakoid-bound ascorbate peroxidase (spot U33), 2-Cys peroxiredoxin (spot U37), physcomitrin (spots U38, U41), N-hydroxycinnamoyl/benzoyltransferase-like protein (spot U42), putative ascorbate peroxidase (spots U45, U46) and peroxiredoxin (spot U50). Proteins involved in protein synthesis, degradation and folding include different family members of the HSP70 family (spots U7, U8, U9, U10, U11, U25, U35 and U52), chloroplast ftsH protease (spot D1) and cullin 3a (spot U27). The up-regulated proteins probable Myb-family transcription factor (spot U2), fibrillarin-like protein (spot U16), probable WRKY transcription factor 52 (spot U22), bZIP transcription factor-like protein (spot U26) and mRNA binding protein precursor-like (spot U44) are all related to transcription. Some signal pathway proteins were also up-regulated, including calcium-dependent protein kinase-like protein (spot U4), ser/thr specific protein kinase-like protein (spot U5) and putative receptor-like protein kinase (spot U14).

## Discussion

In seed plants, the phytohormone ABA controls many developmental and physiological processes via complicated signaling networks that are composed of receptors, secondary messengers, protein kinase, and transcription factors. Transcription factors can meditate protein synthesis in responses to ABA, which is crucial in plant growth and development as well as in plant responses to various stresses. In this study, a broad spectrum of proteins is synthesized following ABA treatment. Detailed analysis of these proteins indicates that the molecular responses to the plant hormone ABA appears to be conserved among higher plant species and bryophytes, as discussed below.

### ABA induced signaling proteins in *P. patens*

Receptor protein kinases (spot U14) are plasma membrane-bound and play an important role in the perception and transmittance of external signals such as ABA, dehydration, high salt and cold treatments [24-26]. Calcium-dependent protein kinases (CDPKs) (spot U4) are implicated as important sensors of Ca^2+ ^flux in plants in response to a variety of environmental stimuli and hormone signals. Depending upon the calcium signature, the extent and duration of CDPK enzyme activation will vary, but will have a direct effect on the phosphorylation status of its downstream targets [27]. Ser/Thr specific protein kinase (spot U5) catalyzes ATP-dependent phosphorylation of serine and threonine residues on target proteins [28]. Phosphorylation by protein kinases is one of the most common and important regulatory mechanisms in signal transduction among all organisms [29]. These results suggested that these same proteins are involved in the ABA signal transduction pathways of *P. patens*, as reported in higher plants [6,7].

### ABA activates the expression of transcriptional regulators

Transcriptional control is a major mechanism whereby cells regulate gene expression. Sequence-specific DNA-binding transcriptional regulators, one class of transcription factors, play an essential role in modulating the rate of transcription of specific target genes [30]. The bZIP transcription factors (spot U26) have been shown to regulate diverse biological processes such as pathogen defense, light and stress signaling, seed maturation and flower development [31]. A group of bZIP proteins have been identified as ABRE-binding factors that activate transcription through this *cis *element [32]. Members of another large family of plant transcription factors, the MYB proteins (spot U2), have also been linked to plant stress responses, including responses to UV light, wounding, anaerobic stress and pathogens [33,34]. WRKY proteins (spot U22) are a novel family of transcription factors that are unique to plants [35]. The WRKY family contains one or two highly conserved WRKY domains characterized by the hallmark heptapeptide WRKYGOK and a zinc-finger structure distinct from other known zinc-finger motifs. Specific WRKY family members show enhanced expression and/or DNA-binding activity following induction by a range of pathogens, defense signals and wounding. These transcriptional regulators have been shown to induce stress-responsive gene expression and protein synthesis and increase plant tolerance to biotic and abiotic stresses in higher plants [36]. Our results suggested that bryophytes, among the earliest land plants, possessed mechanisms of ABA sensing and signal transduction required for stress responses, similar to higher plants.

### ABA induces molecular chaperone synthesis

It is well known that Hsps are induced by ABA in higher plants. Hsps/chaperones can play a crucial role in protecting plants against stress by reestablishing normal protein conformation and thus cellular homeostasis. Five major families of Hsps are conservatively recognized: the Hsp70 (DnaK) family; the chaperonins (GroEL and Hsp60); the Hsp90 family; the Hsp100 (Clp) family; and the small Hsp (sHsp) family [37]. The Hsp70 family (spots U7, U8, U9, U10, U11, U25, U35, U52) represents the most highly conserved of the HSPs, with functional counterparts found in the most primitive of bacteria and the most sophisticated of higher organisms [38]. HSP70 s are involved in almost every step of protein biogenesis [39]. They play essential roles as molecular chaperones assisting the correct folding of nascent polypeptide chains as they emerge from the ribosome, participating in transmembrane protein transport, and limiting cellular damage following stress by their ability to prevent protein aggregation and to restore the function of denatured proteins [40,41]. Luminal binding protein (BiP) (spot U35) is a member of the Hsp70 family that is localized to the endoplasmic reticulum (ER) of eukaryotic cells, where it functions as a chaperone and is believed to support proper protein folding and protein translocation into the ER lumen [42]. HSP is a large protein family that includes HSP90 and HSP60, among others. In our study, different members of Hsp70 subfamily were the major HSPs that were up-regulated by ABA treatment in *P. patens*.

### ABA triggers stress and defense responses

As in higher plants, many defense proteins were up-regulated by ABA signal. In this study, we identified twelve proteins related to defense and cellular protection. Intracellular pathogenesis-related proteins (spot U24) are defined as proteins that are induced by pathogens attack as well as abiotic stimuli. They are powerful defense agents that are currently being use to genetically enhance cereals susceptible to endemic disease and insect pests [43]. In order to combat pathogen infection, plants also produce disease resistance protein (spot U29) that specifically detects the appearance of avirulence products. If they lack corresponding disease resistance proteins, avirulence products can function as virulence factors, subverting host cellular functions through interactions with plant-encoded pathogenicity targets [44]. Lipoxygenases (LOXs) (spot U30) catalyze the formation of fatty acid hydroperoxides involved in response to stresses such as pathogen attack, wounding, water deficit, and anoxia [45]. N-hydroxycinnamoyl/benzoyl transferase (HCBT) (spot U42) catalyses a reaction in the phytoalexin biosynthetic pathway in carnation [46]. Extensin (spots U18, U19) is a hydroxyproline-rich glycoprotein (HRGP) found in the cell walls of higher plants [47]. It is induced by many stimuli such as jasmonic acid, wounding and pathogen attack and appears to play an important part in the plant defense response [48,49]. Proline-rich proteins (spot U15) are also cell wall proteins that actively contribute to the plant defense response [50]. Enzymes involved in antioxidation include ascorbate peroxidase (spots U33, U45, U46), which catalyses the first reaction between H_2_O_2 _and ascorbate in the ascorbate-glutathione cycle, giving rise to monohydroascorbate and H_2_O [51]; and peroxiredoxin (spots. U37, U50), which reduces H_2_O_2 _and detoxifies alkyl hydroperoxides and peroxinitrite [52]. Physcomitrin (spots U38, U41) is a novel plant cytolysin but its biological function is not clear. It is significantly induced by ABA treatment, NaCl, and desiccation [53,54]. and is required Further study is required to determine whether physcomitrin makes a significant contribution in response to extreme environmental conditions in *P. patens*. The identification of a large number of defense proteins implies that ABA could increase the resistance of *P. patens *towards pathogens and abiotic stresses, protecting the plant from oxidative damage.

### ABA inhibits photosynthesis in *P. Patens*

Proteins involved in photosynthesis and carbon assimilation that responded to ABA treatment are represented by the small subunit of Rubisco (spots D9, D13); photosystem II oxygen-evolving complex proteins (spot D4), that catalyze acid-base reactions or that are involved in proton uptake and release [55]; photosystem assembly protein ycf3 (spot D12), which regulates the assembly of photosystem complex proteins. Down-regulation of these proteins suggests that photosynthesis may be inhibited by ABA in *P. patens*. These results were in accord with previous data which showed ABA inhibits photosynthesis in higher plant [8]. Together, our results indicated that ABA-responsive bryophytes underwent physiological changes that had features similar to those of higher plants.

## Conclusions

**Figure 4:**
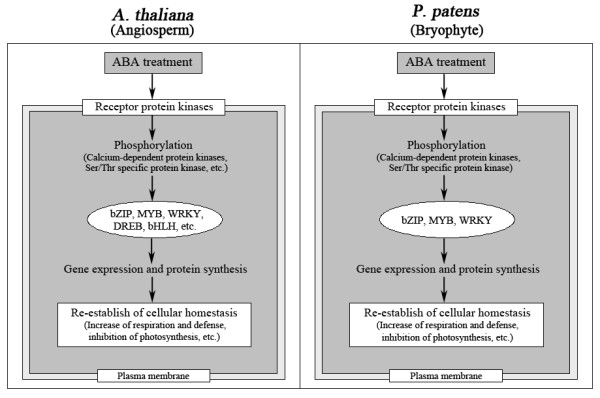
**Comparison of the ABA response processes of *A. thaliana *(Angiosperm) and *P. patens *(Bryophyte)**. ABA signals are perceived by receptor protein kinases and transduced through signaling intermediates to the calcium-dependent protein kinases (CDPKs) and Ser/Thr specific protein kinase, etc. Activated protein kinases phosphorylate and thus activate transcription factors. Subsequently, transcription factors (bZIP factors, MYB, and WRKY, etc) mediate gene expression and protein synthesis in response to ABA treatment. They share three major physiological and biochemical reactions: increase of respiration and defenses, and inhibition of photosynthesis.

Mosses were one of the earliest land plants in evolutionary terms, and have many characteristics in common with seed plants. Therefore, a process common to land plants, such as responding to the plant phytohormone ABA, can be worthwhile to study in the moss species, *P. patens*. In order to investigate global protein changes after treatment with ABA, we have performed a comparative proteome analysis of *P. patens *and revealed a complex regulatory network. Upon the application of ABA, *P. patens *cells can sense ABA and transmit a signal, which, in turn, activates transcriptional factor-mediated metabolic and defensive genes expression. In higher plants, a broad spectrum of genes is also induced by the ABA treatment, and these genes encode not only proteins involved in the protection of cellular structures or in the biosynthesis of protective metabolites but also key regulatory proteins that induce stress-responsive gene expression and protein synthesis and increase plant tolerance to biotic and abiotic stresses. These responses are similar to those seen in the moss *P. patens *in response to ABA. Figure 4 illustrates the ABA response process of angiosperm (*A. thaliana*; from reference 6, 7) and bryophyte (*P. patens*). Based on these results, it seems likely that *P. patens *and higher plants share common receptor protein, protein kinases, transcription factors and physiological and biochemical reactions.

## Methods

### Plant materials

*P. patens *was grown in modified BCD medium containing 0.5% (w/v) glucose and 0.75% (w/v) agar [56]. Gametophores were cultured under the standard conditions (23°C) with a light cycle of 16 h light/8 h darkness and a light intensity of 55 μmol s^-1 ^m^-2^. Three-week-old gametophores were treated with 0 μM or 50 μM ABA for 72 h. For ABA treatment, gametophores were transferred to BCD solid medium supplemented with 50 μM (±)*-cis-trans *ABA. This treatment was carried out three separate times [57]. Gametophores were harvested, frozen in liquid nitrogen and stored at -80°C.

### Chemicals

IPG Drystrip, IPG buffer (pH 3-10), TEMED, ammonium persulfate, acrylamide, SDS and Bis were purchased from GE Healthcare BIO-Science (Uppsala, Sweden); CHAPS, urea, thiourea, iodoacetamide, ammonium bicarbonate, trypsin were purchased from Sigma (St. Louis, MO, USA); DTT and glycine were purchased from Amresco (Solon, OH, USA); ZipTipC 18P™ pipette tips were purchased from Millipore (Bedford, MA, USA).

### Protein extraction and 2-DE

Proteins were extracted using a phenol extraction procedure [54]. The resulting pellets were dissolved in a sample buffer (7 M urea, 2 M thiourea, 4% (w/v) CHAPS, 0.5% (v/v) IPG buffer, 1% (w/v) DTT) at room temperature. 2-D electrophoresis was carried out according to Bjellquist et al. [58]. Dry IPG strips (13 cm long, pH 4-7 linear) were rehydrated for 12 h in 250 μl rehydration buffer containing 800 μg protein samples. Isoelectric focusing was conducted at 20°C with an Ettan IPGphor system (GE Healthcare Amersham Bioscience, Little Chalfont, UK). Focusing was performed and focused strips were then equilibrated as described previously [54]. For the second dimension, the proteins were separated on 15% SDS polyacrylamide gels. Gels were stained with 0.1% Coomassie brilliant blue (CBB) R-250 in 25% ethanol/8% acetic acid and destained in 25% ethanol/8% acetic acid.

### Image and data analysis

The 2-DE gels were scanned at 600 dpi with a UMAX Power Look 2100XL scanner (Maxium Tech Inc., Taiwan, China). The transparency mode was used to obtain a grayscale image. The image analysis was performed with ImageMaster™ 2D Platinum software. To verify the auto detected results, all spots were manually inspected and edited as necessary. After spot detection, quantification, and background subtraction, each analyzed gel was matched individually to the reference gel. To compensate for subtle differences in sample loading, gel staining, and destaining, the volume of each spot (i.e. spot abundance) was normalized as a relative volume. Only those with reproducible changes (quantitative changes more than 1.5-fold in abundance and *t*- test P < 0.05) among three replicates were used for further analysis.

### In-gel digestion and protein identification

Protein spots were manually excised from the gel. In-gel digestion by trypsin and LC-MS/MS were performed according to Wang et al. [54]. All MS/MS samples were analyzed using the TurboSEQUEST program in the BioWorks 3.1 software suite (Thermo), against a database adapted from *P. patens *and *A. thaliana *NCBI protein database, in which each genuine protein sequence was followed by a reversed amino acid sequence. The search parameters included, up to two missed trypsin cleavages with carboxamidomethylation at cysteine. The mass tolerance for precursor ions and fragment ions were considered as 2 amu and 1 amu respectively. Peptides had to be fully tryptically digested and be at least seven amino acids long in the searched databases. The output results were combined together using the software BuildSummary. False positive rate (FPR) was kept less than 5% as the filtration criterion. The FPR was calculated as double of the number of peptides from reversed database divided by the number of peptides from reversed and normal database [59]. An accepted SEQUEST result had a ΔCn score of at least 0.1 (regardless of charge state). Peptides with a +1 charge state were accepted if they were fully tryptic digested and had a cross correlation (Xcorr) of at least 1.9. Peptides with a +2 charge state were accepted if they had an Xcorr ≥2.2. Peptides with a +3 charge state were accepted if they had an Xcorr ≥3.75.

## Authors' contributions

XQW YKH and TYK Conceived and designed the study. XQW Performed the experiments. XQW Analyzed the data. YKH and XQW Contributed reagents/materials/analysis tools. XQW Wrote the paper. All authors read and approved the final manuscript.

## Supplementary Material

Additional file 1 **Supplemental Table S1 Quantitative changes in spot intensities**. The protein abundance is presented as the percentage of the total spot volume associated with each identified spot. The Vol% value of all identified proteins and the data of statistical analysis among three replicates are listed in this file. Click here for file

Additional file 2 **Supplemental Table S2 Peptides sequence matched of ABA responsive proteins**. This file includes the peptides sequence of all identified proteins, total peptide count and unique peptide count which are searched, and charge, XCorr value and score of every peptide. Click here for file
