# Supplementary material for: Conservation between higher plants and the moss Physcomitrella patens in response to the phytohormone abscisic acid: a proteomics analysis
Source: BMC Plant Biol. 2010 Aug 27;10:192. doi: 10.1186/1471-2229-10-192 (PMC2956542; doi:10.1186/1471-2229-10-192)
Supplement: Additional file 1 — Supplemental Table S1 Quantitative changes in spot intensities. The protein abundance is presented as the percentage of the total spot volume associated with each identified spot. The Vol% value of all identified proteins and the data of statistical analysis among three replicates are listed in this file. [file 1471-2229-10-192-S1.DOC]

**Supplemental Table S1** Quantitative changes in spot intensities

| **Spot**  **no.** | **Control**  **Data 1** | **Data 2** | **Data 3** | **Mean**  **value**  **(%Vol)** | **Std** | **ABA treatmet**  **Data 1** | **Data 2** | **Data 3** | **Mean**  **value**  **(%Vol)** | **Std** | ***t*- test**  ***P* value** |
| --- | --- | --- | --- | --- | --- | --- | --- | --- | --- | --- | --- |
| D1 | 0.249 | 0.283 | 0.226 | 0.253 | 0.0287 | 0.132 | 0.148 | 0.162 | 0.147 | 0.0150 | 1.93E-02 |
| D2 | 0.0541 | 0.0572 | 0.0583 | 0.0565 | 0.00218 | 0.0343 | 0.0384 | 0.0391 | 0.0373 | 0.00259 | 1.14E-04 |
| D3 | 0.0885 | 0.0901 | 0.0932 | 0.0906 | 0.00239 | 0.0462 | 0.0485 | 0.0501 | 0.0483 | 0.00196 | 5.24E-05 |
| D4 | 0.203 | 0.252 | 0.231 | 0.229 | 0.0246 | 0.0626 | 0.0683 | 0.0662 | 0.0657 | 0.00288 | 2.93E-03 |
| D5 | 0.544 | 0.523 | 0.492 | 0.520 | 0.0262 | 0.314 | 0.331 | 0.361 | 0.335 | 0.0238 | 1.18E-02 |
| D6 | 0.0727 | 0.0683 | 0.0728 | 0.0713 | 0.00257 | 0.0373 | 0.0332 | 0.0364 | 0.0356 | 0.00215 | 6.09E-05 |
| D7 | 0.529 | 0.536 | 0.584 | 0.550 | 0.0299 | 0.217 | 0.223 | 0.187 | 0.209 | 0.0193 | 3.38E-03 |
| D8 | 0.0992 | 0.107 | 0.134 | 0.113 | 0.0183 | 0.0475 | 0.0427 | 0.0462 | 0.0455 | 0.00248 | 1.17E-02 |
| D9 | 0.102 | 0.0973 | 0.0965 | 0.0986 | 0.00297 | 0.0483 | 0.0441 | 0.0493 | 0.0472 | 0.00276 | 8.24E-04 |
| D10 | 0.0869 | 0.0917 | 0.0876 | 0.0887 | 0.00259 | 0.0293 | 0.0249 | 0.0276 | 0.0273 | 0.00222 | 1.00E-03 |
| D11 | 0.0655 | 0.0643 | 0.0672 | 0.0657 | 0.00146 | 0.0283 | 0.0274 | 0.0235 | 0.0264 | 0.00255 | 1.59E-03 |
| D12 | 0.291 | 0.302 | 0.323 | 0.305 | 0.0163 | 0.204 | 0.172 | 0.213 | 0.196 | 0.0215 | 6.37E-03 |
| D13 | 0.423 | 0.402 | 0.438 | 0.421 | 0.0181 | 0.201 | 0.237 | 0.211 | 0.216 | 0.0186 | 4.65E-03 |
| U1 | 0.0089 | 0.0090 | 0.0092 | 0.0091 | 0.000158 | 0.0747 | 0.0768 | 0.0802 | 0.0772 | 0.00278 | 2.45E-04 |
| U2 | 0.0062 | 0.0063 | 0.0066 | 0.0064 | 0.000235 | 0.0588 | 0.0634 | 0.0623 | 0.0615 | 0.00240\ | 2.83E-04 |
| U3 | 0.0151 | 0.0177 | 0.0182 | 0.017 | 0.00166 | 0.0645 | 0.0693 | 0.0691 | 0.0676 | 0.00272 | 8.21E-05 |
| U4 | 0.0151 | 0.0174 | 0.0197 | 0.0174 | 0.0023 | 0.131 | 0.137 | 0.141 | 0.136 | 0.00503\ | 8.98E-05 |
| U5 | 0.0033 | 0.0033 | 0.0031 | 0.0032 | 0.000111 | 0.114 | 0.125 | 0.127 | 0.122 | 0.007 | 5.84E-04 |
| U6 | 0.0039 | 0.0044 | 0.0042 | 0.0042 | 0.000240 | 0.183 | 0.211 | 0.223 | 0.206 | 0.0205\ | 1.69E-03 |
| U7 | 0.0493 | 0.0463 | 0.0457 | 0.0471 | 0.00193 | 0.911 | 0.953 | 0.965 | 0.943 | 0.0284 | 1.90E-04 |
| U8 | 0.0866 | 0.0892 | 0.0923 | 0.0894 | 0.00285 | 0.314 | 0.328 | 0.359 | 0.334 | 0.0230\ | 1.14E-03 |
| U9 | 0.131 | 0.125 | 0.154 | 0.137 | 0.0153 | 0.221 | 0.225 | 0.263 | 0.236 | 0.0232 | 1.51E-03 |
| U10 | 0.0328 | 0.0276 | 0.0291 | 0.0298 | 0.00268 | 0.0587 | 0.0549 | 0.0592 | 0.0576 | 0.00235 | 9.86E-04 |
| U11 | 0.0242 | 0.0231 | 0.0283 | 0.0252 | 0.00274 | 0.0783 | 0.0825 | 0.0794 | 0.0801 | 0.00218 | 9.75E-04 |
| U12 | 0.0573 | 0.0524 | 0.0583 | 0.056 | 0.00316 | 0.125 | 0.103 | 0.0994 | 0.109 | 0.0139 | 1.04E-02 |
| U13 | 0.0523 | 0.0471 | 0.0491 | 0.0495 | 0.00262 | 0.151 | 0.166 | 0.171 | 0.163 | 0.0104 | 2.06E-03 |
| U14 | 0.0719 | 0.0748 | 0.0707 | 0.0725 | 0.00211 | 0.439 | 0.395 | 0.423 | 0.419 | 0.0223 | 7.96E-04 |
| U15 | 0.0391 | 0.0354 | 0.0382 | 0.0376 | 0.00193 | 0.0934 | 0.0908 | 0.0965 | 0.0936 | 0.00285 | 2.27E-04 |
| U16 | 0.0032 | 0.0030 | 0.0031 | 0.0031 | 0.000115 | 0.0883 | 0.0922 | 0.0901 | 0.0902 | 0.00195 | 9.38E-05 |
| U17 | 0.0302 | 0.0312 | 0.0354 | 0.0323 | 0.00276 | 0.0521 | 0.0553 | 0.0562 | 0.0545 | 0.00215 | 9.46E-04 |
| U18 | 0.0276 | 0.0321 | 0.0312 | 0.0303 | 0.00238 | 0.0513 | 0.0548 | 0.0563 | 0.0541 | 0.00257 | 4.26E-04 |
| U19 | 0.0221 | 0.0213 | 0.0254 | 0.0229 | 0.00217 | 0.0726 | 0.0719 | 0.0773 | 0.0739 | 0.00294 | 3.91E-05 |
| U20 | 0.0455 | 0.0418 | 0.0463 | 0.0445 | 0.00240 | 0.248 | 0.217 | 0.253 | 0.239 | 0.0195 | 1.28E-03 |
| U21 | 0.0892 | 0.0853 | 0.0901 | 0.0882 | 0.00255 | 0.201 | 0.163 | 0.194 | 0.186 | 0.0202 | 5.46E-03 |
| U22 | 0 | 0 | 0 | 0 | 0 | 0.0685 | 0.0724 | 0.0673 | 0.0694 | 0.00267 | 2.46E-04 |
| U23 | 0.0348 | 0.0304 | 0.0335 | 0.0329 | 0.00226 | 0.722 | 0.759 | 0.718 | 0.733 | 0.0226 | 2.08E-04 |
| U24 | 0 | 0 | 0 | 0 | 0 | 0.133 | 0.145 | 0.131 | 0.136 | 0.00757 | 5.13E-04 |
| U25 | 0.189 | 0.227 | 0.225 | 0.214 | 0.0214 | 0.557 | 0.523 | 0.562 | 0.547 | 0.0212 | 1.94E-03 |
| U26 | 0.286 | 0.278 | 0.234 | 0.266 | 0.028 | 0.411 | 0.389 | 0.425 | 0.408 | 0.0181 | 1.44E-02 |
| U27 | 0.0975 | 0.108 | 0.0993 | 0.102 | 0.00562 | 0.203 | 0.193 | 0.187 | 0.194 | 0.00808 | 2.39E-03 |
| U28 | 0.0403 | 0.0364 | 0.0396 | 0.0388 | 0.00208 | 0.0716 | 0.0673 | 0.0661 | 0.068 | 0.00289 | 1.35E-03 |
| U29 | 0.108 | 0.0984 | 0.134 | 0.114 | 0.0184 | 0.231 | 0.211 | 0.254 | 0.232 | 0.0215 | 3.40E-04 |
| U30 | 0.0498 | 0.0511 | 0.0531 | 0.0513 | 0.00166 | 0.0952 | 0.0977 | 0.102 | 0.0983 | 0.00344 | 2.39E-04 |
| U31 | 0.204 | 0.226 | 0.242 | 0.224 | 0.0191 | 0.316 | 0.328 | 0.367 | 0.337 | 0.0267 | 1.73E-03 |
| U32 | 0.0604 | 0.0573 | 0.0625 | 0.0601 | 0.00262 | 0.103 | 0.117 | 0.125 | 0.115 | 0.0111 | 6.29E-03 |
| U33 | 0.382 | 0.349 | 0.394 | 0.375 | 0.0233 | 0.604 | 0.593 | 0.552 | 0.583 | 0.0274 | 7.52E-03 |
| U34 | 0.0099 | 0.0137 | 0.0097 | 0.0111 | 0.00223 | 0.147 | 0.138 | 0.149 | 0.145 | 0.00586 | 6.09E-04 |
| U35 | 0.173 | 0.164 | 0.178 | 0.172 | 0.00709 | 0.556 | 0.547 | 0.582 | 0.562 | 0.0182 | 1.61E-04 |
| U36 | 0.0413 | 0.0383 | 0.0432 | 0.0409 | 0.00247 | 0.237 | 0.201 | 0.256 | 0.231 | 0.0279 | 2.96E-03 |
| U37 | 0.0404 | 0.0375 | 0.0413 | 0.0397 | 0.00199 | 0.267 | 0.238 | 0.293 | 0.266 | 0.0275 | 2.12E-03 |
| U38 | 0.106 | 0.128 | 0.144 | 0.126 | 0.0191 | 0.214 | 0.248 | 0.273 | 0.245 | 0.0296 | 1.30E-03 |
| U39 | 0.0727 | 0.0698 | 0.0735 | 0.072 | 0.00195 | 0.304 | 0.283 | 0.332 | 0.306 | 0.0246 | 1.57E-03 |
| U40 | 0 | 0 | 0 | 0 | 0 | 0.102 | 0.118 | 0.129 | 0.116 | 0.0136 | 2.25E-03 |
| U41 | 0.0964 | 0.126 | 0.0993 | 0.107 | 0.0163 | 0.471 | 0.469 | 0.482 | 0.474 | 0.007 | 5.44E-04 |
| U42 | 0.0486 | 0.0438 | 0.0498 | 0.0474 | 0.00317 | 0.118 | 0.109 | 0.142 | 0.123 | 0.0171 | 6.04E-03 |
| U43 | 0 | 0 | 0 | 0 | 0 | 0.182 | 0.225 | 0.206 | 0.204 | 0.0215 | 1.84E-03 |
| U44 | 0.148 | 0.139 | 0.174 | 0.154 | 0.0182 | 0.264 | 0.233 | 0.245 | 0.247 | 0.0156 | 9.35E-03 |
| U45 | 0.342 | 0.395 | 0.411 | 0.383 | 0.0361 | 1.15 | 1.23 | 1.02 | 1.133 | 0.106 | 4.45E-03 |
| U46 | 0.0451 | 0.0483 | 0.0501 | 0.0478 | 0.00253 | 0.14 | 0.118 | 0.153 | 0.137 | 0.0177 | 6.18E-03 |
| U47 | 0.0253 | 0.0293 | 0.0281 | 0.0276 | 0.00205 | 0.491 | 0.463 | 0.458 | 0.471 | 0.0178 | 3.28E-04 |
| U48 | 0.0553 | 0.0543 | 0.0589 | 0.0562 | 0.00242 | 0.394 | 0.356 | 0.379 | 0.376 | 0.0191 | 5.56E-04 |
| U49 | 0.0993 | 0.126 | 0.119 | 0.115 | 0.0138 | 0.619 | 0.583 | 0.628 | 0.61 | 0.0238 | 7.63E-04 |
| U50 | 0.208 | 0.233 | 0.245 | 0.229 | 0.0189 | 0.352 | 0.365 | 0.331 | 0.349 | 0.0172 | 1.04E-02 |
| U51 | 0.107 | 0.0983 | 0.112 | 0.106 | 0.00693 | 0.397 | 0.438 | 0.407 | 0.414 | 0.0214 | 1.31E-03 |
| U52 | 0.0103 | 0.0107 | 0.0142 | 0.0117 | 0.00215 | 0.0293 | 0.0271 | 0.0312 | 0.0292 | 0.00205 | 1.01E-03 |
