# Supplementary material for: Conservation between higher plants and the moss Physcomitrella patens in response to the phytohormone abscisic acid: a proteomics analysis
Source: BMC Plant Biol. 2010 Aug 27;10:192. doi: 10.1186/1471-2229-10-192 (PMC2956542; doi:10.1186/1471-2229-10-192)
Supplement: Additional file 2 — Supplemental Table S2 Peptides sequence matched of ABA responsive proteins. This file includes the peptides sequence of all identified proteins, total peptide count and unique peptide count which are searched, and charge, XCorr value and score of every peptide. [file 1471-2229-10-192-S2.DOC]

**Supplemental Table S2** Peptides sequence matched of ABA responsive proteins

| **Spot**  **no.** | **Sequence** | **TPC/**  **UPC^a^** | **Charge** | **XCorr** | **Score** |
| --- | --- | --- | --- | --- | --- |
| D1 | LVAYHEAGHALVGALMPEYDPVAK  VAEEVIFGDENVTTGASNDFMQVSR  TPGFTGADLQNLMNEAAILAAR  FQEVPETGVSFADVAGADQAK  SYLENQMAVALGGR  YSEFLNAVK | 13/6 | 2  2  2  2  2  1 | 2.315  4.3712  4.827  3.671  4.6406  2.096 | 324.3  1180.9  947.9  478.8  1884.7  483.6 |
| D2 | KADGAHELLFGVINYKDK | 2/1 | 2 | 2.2796 | 613.1 |
| D3 | EGSITSIQAVYVPADDLTDPAPATTFAHLDATTVLSR GIYPAVDPLDSTSTMLQPWIVGEEHYETAQGVK  GFQMILSGELDSFPEQAFYLVGNIDEATAK.A  IFNVLGEPVDNLGPVDASTTFPIHR  GQNTAGQEINVTCEVQQLLGNNK  GMDVVDTGAALSVPVGEATLGR  MPSAVGYQPTLSTEMGTLQER  YKELQDIIAILGLDELSEEDR  VALVYGQMNEPPGAR  DVNKQDVLLFIDNIFR  ISQIIGPVLDVTFPPGK  AHGGVSVFGGVGER  FVQAGSEVSALLGR  VGLTALTMAEYFR  AVAMSATDGLMR  IGLFGGAGVGK  SAPAFTQLDTK  VINEENISESK | 36/18 | 3  3  3  2  2  2  2  2  2  2  2  2  2  2  2  2  2  1 | 4.3965  4.7277  4.9198  5.0274  5.8726  5.5158  4.4732  5.2727  4.2609  5.7503  2.9683  3.9208  4.8996  4.5577  2.9586  3.116  2.9911  1.965 | 524.5  714  879.5  1194.8  2018.7  971.6  1048.9  1704.3  1003.4  2216.5  536.9  1114.2  2585.6  1537.2  1521  1125.9  455.7  329.9 |
| D4 | FKEEDGIDYAAVTVQLPGGER  GSSFLDPK | 2/2 | 2  2 | 5.9024  2.2722 | 2257.8  440.4 |
| D5 | MNIAAWASKEEEENK | 2/1 | 2 | 2.3149 | 234.3 |
| D6 | CLEQIFETRQSLR | 2/1 | 2 | 3.3236 | 1075.7 |
| D7 | EGAITIIGGGDSVAAVEK  AHSSTAGIAEYVGK | 4/2 | 2  2 | 2.965  3.3803 | 309.6  1207.5 |
| D8 | GGAFTGEISADQLVDVGVK  IIYGGSVNGANSAELAK  QEDIDGFLVGGASLK  VASPQQAQEVHSAIR  VVIAYEPVWAIGTGK  RHVIGESNSTVGK  IEVSAQNSWVGK  HVIGESNSTVGK  WVIQGHSER  ISPEVSSATR | 15/10 | 2  2  2  2  2  2  2  2  2  2 | 5.439  5.1027  4.4733  4.0479  4.4079  4.1968  3.8417  2.9156  2.8553  2.4563 | 1970  1134.2  1855  1356.1  1741.4  1375.7  1539.9  943.5  1462.7  729.8 |
| D9 | QVDYMIQQNLIPCIEFDTVGAVSR  FETFSYLPPLSDDQIAR  TNFSGSGSSGYYDGR  KQVQICGFLVARPN  LPMFGCTDSSQVLR | 9/5 | 2  2  2  2  2 | 4.1479  3.0247  4.7998  4.155  4.2037 | 406.4  372.5  1517.4  1043.4  1370.1 |
| D10 | NNLGDGELCSVSGSSRNVRFPR | 4/1 | 2 | 2.3988 | 226.8 |
| D11 | ALQESLASELAAR | 2/1 | 2 | 3.8547 | 2152.3 |
| D12 | GEQAIQQGDSETSEAWFNQAADYWK  DGMSAQSEGEYAEALQNYYEAMR  QAIALAPSNYIEAQNWLK  NPSLPQAFNNMAVICHYR  SYILYNIGLIHTSNGEHAK  NDNFIDK | 12/6 | 2  2  2  2  2  1 | 6.6986  5.9696  4.8984  4.7103  3.6077  2.1138 | 2125.7  1596  1036.2  725.1  710.8  347.7 |
| D13 | QVDYMIQQNLIPCIEFDTVGSVSR  FETFSYLPPLTDDQIAK  TNFSGAGSSGYYDGR  LPMFGCTDSSQVLR  KQVQICGFLVSRPN  QVQICGFLVSRPN  SQFPSCWVR  VLGFDSKK  YWTMWKL  VLGFDSK | 62/10 | 2  2  2  2  2  2  2  2  1  1 | 4.5437  4.4478  4.6162  4.0752  5.1606  3.6707  2.5296  2.4567  1.9242  1.9118 | 208.7  695.5  2046.8  1214.5  1554.7  1803.8  691.6  514.5  600.2  476.4 |
| U1 | DKMADIMRYMNR | 2/1 | 2 | 2.6251 | 736.1 |
| U2 | TAVAASLPRSSGIISAPK | 2/1 | 2 | 2.3499 | 170.1 |
| U3 | TTGIVLDSGDGVTHTVPIYEGYALPHAILR  DLYGNIVLSGGSTM*FPGIADR  VAPEEHPVLLTEAPLNPK  YPIEHGVVTNWDDMEK  SYELPDGQVITIGAER  SEYDESGPSIVHR  AGFAGDDAPR  EITALAPSSMK  DLTDALMK | 18/9 | 3  2  2  2  2  2  1  2  1 | 5.4151  5.3666  4.4959  5.0163  4.6647  4.0468  2.1313  2.8888  1.9856 | 1228.2  1699.8  1057  1420.3  1423.3  2242.1  388.1  811.6  124.1 |
| U4 | DLM*EAADIDK | 2/1 | 2 | 2.6724 | 911.6 |
| U5 | EFKSEVEILSK | 2/1 | 2 | 2.3013 | 824.7 |
| U6 | RRVEDGGAEIGR | 2/1 | 2 | 2.5067 | 719.8 |
| U7 | AVITVPAYFNDSQR  QFAAEEISAQVLR  AKFEELCSDLLDR | 7/3 | 2  2  2 | 2.8124  4.6742  4.1466 | 412.8  2090.8  2230.8 |
| U8 | AVITVPAYFNDSQR  QFAAEEISAQVLR  AKFEELCSDLLDR | 7/3 | 2  2  2 | 2.8124  4.6742  4.1466 | 412.8  2090.8  2230.8 |
| U9 | SINPDEAVAYGAAVQAAILSGEGNEK  NVLIFDLGGGTFDVSLLTIEEGIFEVK  IINEPTAAAIAYGLDKK  VQQLLQDFFNGKELCK  ATAGDTHLGGEDFDNR  IINEPTAAAIAYGLDK  NAVVTVPAYFNDSQR  ARFEEMNMDLFR  VQQLLQDFFNGK  DAGVIAGLNVLR  VEIIANDQGNR  MVNHFVQEFK | 28/12 | 2  2  2  2  2  2  2  2  2  1  1  2 | 7.2387  3.253  4.8046  4.7018  4.2696  5.5501  3.3869  2.8603  4.4495  2.017  2.0431  3.3882 | 1959.7  631.5  1062  1178.3  1526.6  1391.8  472.7  488.9  2427  `237.2  285.2  1386.1 |
| U10 | MDKSTVHDVVLVGGSTR  IINEPTAAAIAYGLDKK  ATAGDTHLGGEDFDNR  IINEPTAAAIAYGLDK  NAVVTVPAYFNDSQR  ARFEELNMDLFRK  TTPSYVAFTDSER  ARFEELNMDLFR  FEELNMDLFR  ITITNDKGR | 23/10 | 2  2  2  2  2  3  2  2  2  2 | 3.3917  4.6595  3.8509  5.4288  3.9558  4.0064  3.4684  4.3531  2.7849  2.5252 | 1057.6  1089.8  1459.2  1589.4  396.6  1182.2  1136.9  1169.7  920.8  738.5 |
| U11 | SINPDEAVAYGAAVQAAILSGEGNEK  ATAGDTHLGGEDFDNR  IINEPTAAAIAYGLDKK  IINEPTAAAIAYGLDK  NQVAMNPTNTVFDAK  NAVVIVPAYFNDSQR  ARFEELNMDLFR  VEIIANDQGNR  FEELNMDLFR  ITITNDKGR  DNNLLGK | 37/11 | 2  2  2  2  2  2  2  2  2  2  1 | 5.3171  4.4916  4.9606  5.0566  4.6366  2.2955  4.1866  3.8924  3.9752  2.8227  1.9375 | 1384.4  1481.1  1111.1  1469.2  1499.6  212.5  1178.8  1775.1  1505.5  984.8  542.8 |
| U12 | LFQVEAKSVIIAAK | 2/1 | 2 | 2.4051 | 700.9 |
| U13 | ALEVETPIAVSYVESLEDKNAK  ALEVETPIAVSYVESLEDK  ENAAIPFEADLPEYVEPK  FGYDGDFSLESVKEFGEK  EVAAPVLEFFSLSGEK  VTITGFPTVIFFPAGK  AATILKDDGAVLAK  QFLLFAGPEEYAK  FGYDGDFSLESVK  SEDIPETNDEPVK  SIFESDTNK | 19/11 | 2  2  2  2  2  2  2  2  1  2  1 | 4.6428  5.0069  3.9685  3.0982  3.5408  4.3488  4.2923  3.2571  2.4248  3.2022  2.1136 | 1004.1  1069.3  687.9  682.3  793.4  787.2  1470.1  899.7  527.2  530.1  536.9 |
| U14 | EYLVNM*TDAQFALR | 3/1 | 2 | 3.0014 | 924.4 |
| U15 | LLEEPKPEIPTFPELPKPEMPK | 3/1 | 2 | 2.8466 | 342.1 |
| U16 | M*LVGM*VDVIFSDVAQPDQAR | 2/1 | 2 | 2.7115 | 338.7 |
| U17 | KENPDGDEPQNPTLVR | 2/1 | 2 | 2.2744 | 606.3 |
| U18 | AGIVPILYK | 2/1 | 1 | 1.9753 | 459.2 |
| U19 | KEVPPPVPVYKPPPK | 2/1 | 2 | 3.0761 | 641.7 |
| U20 | GADM*VFVTAGM*GGGTGSGAAPIIAGVAK  SPTGLSQGSNGSAINIPSFLR  GLGAGGNPEIGCSAAEESK  EAALSAIQSPLLDVGIER | 6/4 | 2  2  2  2 | 5.1804  6.2738  4.2209  5.3085 | 2276.3  1580.6  1222.6  1702.5 |
| U21 | EAGALLSYDPNLR | 2/1 | 2 | 2.7071 | 483.9 |
| U22 | FDLQPSAFKNMLNLRLLK | 2/1 | 2 | 2.4773 | 342 |
| U23 | EAGALLSYDPNLR | 2/1 | 2 | 3.3321 | 936.1 |
| U24 | VLHFGPAIPQAGAAK | 2/1 | 2 | 3.5191 | 1085.5 |
| U25 | SINPDEAVAYGAAVQAAILSGEGNEK  VQQLLQDFFNGK | 3/2 | 2  2 | 6.784  3.1722 | 1910  1273.7 |
| U26 | IIDGPPEILMERR | 4/1 | 2 | 2.7329 | 746 |
| U27 | LSWQTNMGTADIKAIFGK | 2/1 | 2 | 2.4912 | 459.6 |
| U28 | WAVLVAGSSGYGNYR | 2/1 | 2 | 3.1382 | 548.8 |
| U29 | TTLSANIFKSQSVRR | 2/1 | 2 | 2.3459 | 451.1 |
| U30 | DTWTFESQALPTDLVAR  IFFQNTAYLPDETPASLK | 2/2 | 2  2 | 2.2063  4.5302 | 342  825.1 |
| U31 | GGAFTGEISAEQLADAGVK  VASPQQAQEVHAAIR  HVIGETDAMIGKK  RHVIGETDAMIGKK  WVIQGHSER | 19/5 | 2  2  2  2  2 | 3.6106  4.6004  4.1348  4.0944  3.0708 | 543.5  1330.1  1115.9  910.6  1470.8 |
| U32 | VVIAYEPVWAIGTGK  GLGLIACVGELLEER  GPEFATICNAVTAK  WVIQGHSER | 8/4 | 2  2  2  2 | 4.0984  4.5338  4.07  2.6375 | 1688.1  1589.1  1542.9  1184.9 |
| U33 | ELSDSMKK | 2/1 | 1 | 2.1444 | 567.8 |
| U34 | TPDTKSVEVIETGKIK | 2/1 | 2 | 2.9708 | 557.5 |
| U35 | IINEPTAAAIAYGLDK  IINEPTAAAIAYGLDKK | 2/2 | 2  2 | 2.4748  2.3665 | 700.5  519.7 |
| U36 | HVVIVDDLVQSGGTLIECQK | 2/1 | 2 | 2.287 | 544.2 |
| U37 | GLFIIDK | 2/1 | 1 | 2.1619 | 528.2 |
| U38 | TLQNIVEGITGVDRK  GTVGTFSYYIQDEDK  TLQNIVEGITGVDR  INAQEALLFSAR | 7/4 | 2  2  2  2 | 401457  3.6602  4.264  4.5351 | 1378.2  1030.6  1194.3  2215.2 |
| U39 | DNISVVVVDLKPR | 4/1 | 2 | 2.3401 | 1179.9 |
| U40 | M*ATGGKVSFKVTLTSDPK | 2/1 | 2 | 2.7376 | 351.1 |
| U41 | NLTDYTLENLGVYFNSGSSDR  TLQNIVEGITGVDRK  GTVGTFSYYIQDEDK  TLQNIVEGITGVDR  INAQEALLFSAR  KIAIGFK | 24/6 | 2  2  2  2  2  1 | 6.1485  4.741  3.9903  4.3918  4.7378  2.4322 | 1632.2  1482.8  1393.3  1759.2  2749.6  607.5 |
| U42 | YVPLVVQSFFDHHK | 2/1 | 2 | 2.4275 | 279.9 |
| U43 | GTM*TTTHSYTGDQR  AAALNIVPTSTGAAK | 3/2 | 2  2 | 2.8841  3.324 | 1220.4  532.4 |
| U44 | QFLFISSAGIYK  NMHFYAEPR | 3/2 | 2  2 | 2.956  2.6487 | 679.3  948.6 |
| U45 | QM*GLTDKDIVVLSGAHTLGR  SGFEGAWTPNPLR | 5/2 | 2  2 | 3.688  3.5376 | 509.9  980.7 |
| U46 | QM*GLTDKDIVVLSGAHTLGR  LAWHGSGTYDQESK  SGFEGAWTPNPLR | 7/3 | 2  2  2 | 4.3594  3.8268  3.4649 | 729  1408.4  1056.4 |
| U47 | EHDATEQEM*DDEAADHKEGK | 2/1 | 2 | 2.2437 | 310.9 |
| U48 | FRQILTNLMGNSIK | 2/1 | 2 | 2.924 | 949 |
| U49 | NCFAIASDRR  NCFAIASDR | 6/2 | 2  2 | 2.484  3.5887 | 551.7  1395.3 |
| U50 | LLGLSCDDVQSHK | 2/1 | 2 | 2.5189 | 1274.1 |
| U51 | HVVIVDDLVQSGGTLIECQK | 2/1 | 2 | 2.5627 | 448.5 |
| U52 | NAVVTVPAYFNDSQR  IINEPTAAAIAYGLDK | 2/2 | 2  2 | 2.6025  3.2748 | 193.6  586.8 |

^a^ TPC/UPC stands for total peptide count/unique peptide count which are searched.

* Methylation sites
